# Investigating the Value of Abatacept in the Treatment of Rheumatoid Arthritis: A Systematic Review of Cost-Effectiveness Studies

**DOI:** 10.1155/2013/256871

**Published:** 2013-05-30

**Authors:** Kostas Athanasakis, Ioannis Petrakis, John Kyriopoulos

**Affiliations:** Department of Health Economics, National School of Public Health, 196 Alexandras Avenue, 11521 Athens, Greece

## Abstract

*Background*. Rheumatoid arthritis is a progressive inflammatory disease that affects greatly patients' quality of life and demands for aggressive management early on during the course of the disease. The discovery of biologics has equipped rheumatologists with evolutionary treatment tools but has also impacted greatly management costs. *Objectives*. To conduct a systematic review in order to evaluate the cost effectiveness of abatacept in the treatment of moderate to severe rheumatoid arthritis. *Methods*. Pubmed, the International Society for Pharmacoeconomics and Outcomes Research Outcomes Research Digest, the National Health System Economic Evaluation Database, and the Database of Abstracts of Reviews of Effects were searched. *Results*. In total 301 studies were identified and 42 met the inclusion criteria. Half of the selected studies evaluated abatacept in the treatment of rheumatoid arthritis, after failure of or intolerance to tumor necrosis factor alpha inhibitors. Of those, 82% were in favor of abatacept as a cost-effective or dominant strategy versus varying alternatives, whereas 18% favored other treatments. *Conclusion*. The majority of evidence from the published literature supports that abatacept can be a cost-effective alternative in the treatment of moderate to severe rheumatoid arthritis, especially in patients that have demonstrated inadequate response or intolerance to anti-TNF agents or conventional disease modifying antirheumatic drugs.

## 1. Introduction

Rheumatoid arthritis (RA) is a systemic inflammatory disease that presents itself in multiple joints of the musculoskeletal system. Symptoms include joint swelling, redness, and pain with gradual joint deformity in some cases. Due to its pathophysiology, RA causes not only significant morbidity and progressive loss of quality of life, but also carries a substantial economic burden, both for the individuals as well as for the society as a whole, since it is associated with high intense short- and long-term healthcare resource utilizations due to its increased prevalence and disability potential [1].

Over the last two decades, researchers have equipped rheumatologists with revolutionary therapeutic options. However, these changes have not been fully brought into effect in many European countries and other parts of the world [2, 3]. Disease-modifying antirheumatic drugs (DMARDs) represent the mainstay of RA management. Corticosteroids and nonsteroidal anti-inflammatory drugs (NSAIDs) are also employed, mainly in the short-term, arthritis-related, symptomatic mitigation. Recently, several disease modifying biologic agents have been licensed for the management of RA, alone or in combination with methotrexate (MTX). These agents include abatacept (ABA), adalimumab (ADA), anakinra (ANA), certolizumab pegol (CER), etanercept (ETA), golimumab (GOL), infliximab (INF), rituximab (RTX), and tocilizumab (TOC). 

Abatacept is a selective modulator that blocks T-cell activation. It has a marketing authorisation for use in combination with MTX for the treatment of moderate to severe active rheumatoid arthritis in adults whose disease has responded inadequately to previous therapy with one or more DMARDs, including MTX or a tumour necrosis factor (TNF) inhibitor [4]. Its unique mode of action results in inhibition of the inflammatory cascade by preventing activation of T-cells through binding to the natural ligands CD80 and CD86 not allowing their interaction with CD28 on the T-lymphocyte [5]. 

The plethora of therapeutic options available and the variability of patient subgroups that represent target populations for each medication have led to the publication of a number of guidelines and treatment algorithms internationally. Among the agencies that have published such guidance, the National Institute of Clinical Excellence (NICE) has issued several guidelines on the use of biologics in RA [6], producing a rather complex prescribing regime [7]. According to the NICE recommendations, TNF antagonists are the only class of biologic agents that can be used as first line therapy, limiting the use of other agents with different mode of action. Biologic DMARD sequencing cannot be pinpointed accurately after intolerance to or failure of a TNF antagonist, and thus treatment regimens should be based not only to the licensed indications, but to the cost-effectiveness data available [6]. The latter has become a global objective, especially today, since scarcity of resources is more than evident.

In light of the aforementioned, the purpose of the present study was to conduct a systematic review of cost-effectiveness studies of abatacept in order to evaluate the role of this agent in the treatment of moderate to severe rheumatoid arthritis, specializing to treatment algorithms after previous inadequate response or intolerance to disease modifying antirheumatic drugs (DMARDs) and/or TNF inhibitors. 

## 2. Materials and Methods

### 2.1. Overview

The literature search was extended to a wide range of databases, in order to include as much and as recent information as possible. The databases that were searched included Pubmed, the International Society for Pharmacoeconomics and Outcomes Research (ISPOR) Outcomes Research Digest, the National Health System Economic Evaluation Database (NHS EED), and the Database of Abstracts of Reviews of Effects (DARE). Overlapping hits were included once. The literature search was not limited by study type or publication year; however, study selection was limited to articles written in English (inclusion and exclusion criteria are listed in detail later). For economic evaluation data, the outcomes included cost effectiveness and related types of analyses (including cost consequence, cost of treatment, and budget impact studies).

### 2.2. Preliminary Search

A preliminary database search was conducted in order to challenge the systematic review strategy, focusing on the keywords to be used and the need for limiting the main literature search. Furthermore, it provided an estimate of the size of the literature available on the specific topic.

### 2.3. Main Search

The main search was carried out in August 2012. Slightly different strategies were employed for each database searched due to inherent limitations pertaining to the type and classification of information. An initial search using the keywords “abatacept, cost effectiveness, and rheumatoid arthritis” was carried out in Pubmed. The Pubmed search was enriched by selecting the “related citations” option to each one of the initial results. The study that was selected as the basis for related citations was one containing all keywords in the title and was deemed the most relevant to the objective [8]. The ISPOR Outcomes Research Digest was searched by using the in-title term “abatacept” in the “rheumatoid arthritis” disease/disorder section. Additional searches employed the same strategy; however, the disease/disorder section selected was “arthritis” and “Muscular-skeletal Disorders (including Tunnel Syndrome).” No other limits were used. DARE and NHS EED databases were searched through the Centre for Reviews and Dissemination. “Abatacept” and “rheumatoid arthritis” were searched as keywords to the titles of published studies, without limiting databases or record types. 

### 2.4. Study Selection

Two members of the review team assessed the titles of the identified results independently. All relevant hits were then cross-checked against the inclusion and exclusion criteria. Studies that were identified by only one of the reviewers were examined in a separate meeting, in order for an inclusion decision to be reached. 

### 2.5. Inclusion Criteria

The criteria which all initial results were examined against are listed as follows:

- studies comparing abatacept to other biologics or conventional therapies in rheumatoid arthritis;
- studies included both systematic reviews as well as stand-alone economic evaluation studies;
- types of costs included direct and indirect costs—effectiveness measures included all life expectancy and quality of life outcome measures;
- studies focusing on adult patients.

### 2.6. Exclusion Criteria

Exclusion criteria were as follows:

- studies comparing abatacept to other treatments in different indications than rheumatoid arthritis;
- studies not reporting cost data—comparisons to other treatment options only on clinical effectiveness grounds were excluded;
- studies not specifying a time horizon for costs or results (for standalone economic evaluation studies);
- studies comparing treatments in juvenile rheumatoid arthritis;
- studies with no research abstract and/or author details available;
- articles commenting on previous research studies;
- studies that did not meet the quality assessment criteria.

### 2.7. Data Extraction and Quality Assessment

All relevant study information was extracted into predesigned tables. Fields included study information and origin, study type, type of economic evaluation, comparing treatments, results, and comments. Studies that were identified in multiple databases were reported once. The quality of independent economic evaluation studies was evaluated in accordance to the Centre for Reviews and Dissemination set of guidelines [9].

### 2.8. Analysis of Findings

All relevant studies are presented and analyzed according to the line of therapy or treatment algorithm in rheumatoid arthritis. The main analyses focused on (a) abatacept use after failure of traditional DMARD therapies and (b) abatacept use after an inadequate response (IR) to TNF-a inhibitor therapy. All other relevant pharmacoeconomic studies which met the inclusion criteria but could not be accurately classified in one of the previous approaches are presented and analyzed in a separate section (c).

## 3. Results

In total, 301 studies were identified. The initial Pubmed search produced 19 hits and the secondary search produced another 197 results. The CRD database produced 8 hits and finally the ISPOR database revealed 50, 24, and 3 matches for “rheumatoid arthritis,” “arthritis,” and “Muscular-skeletal Disorders (including Tunnel Syndrome)” disease sections, respectively. After the inclusion and exclusion criteria were applied, 42 studies were finally selected. The majority of rejected studies were due to lack of cost data, failure to include abatacept as a comparator to other biologic agents, and failure to include RA as a treatment indication. Other reasons for exclusion included failure to meet the quality assessment criteria (1 study) and comparison of different biologic sequences, where results were presented for single agents (other than abatacept) and not the entire sequencing strategy containing abatacept (2 studies).

### 3.1. Abatacept after Conventional DMARD

In total 9 studies on the cost effectiveness of abatacept in the treatment of rheumatoid arthritis after failure of or intolerance to conventional DMARDs were identified. All retrieved publications followed the cost-effectiveness analysis. Two of the previous also presented budget impact data for the agents under comparison. The results are presented in detail in Table 1.

### 3.2. Abatacept after TNF-Antagonist

In total 21 studies on the cost effectiveness of abatacept in the treatment of rheumatoid arthritis after failure of or intolerance to TNF-a inhibitors were identified. Apart from the “classic” economic evaluations in the form of cost-effectiveness analysis, the results under this section included other approaches, such as cost of therapy and budget impact analysis. The results are presented in detail in Table 2.

### 3.3. Other Pharmacoeconomic Studies Involving Abatacept

An additional 12 pharmacoeconomic studies relevant to the role of abatacept in the treatment of rheumatoid arthritis were identified but could not be included into one of the two major treatment pathways mentioned earlier. Those studies mainly included abatacept use after conventional DMARD therapy and anti-TNF replacement or studies that failed to specify the line or sequence of treatment and simply compared the cost effectiveness of abatacept against other modes of therapy. Those results are presented in Table 3.

### 3.4. Summary of Findings

A wide range of economic evaluation and cost-analysis studies were identified with several comparators under investigation. Abatacept mainly appears to have been studied after failure of a TNF inhibitor. In regards to the economic evaluation studies, abatacept's cost effectiveness appears to range within the acceptable cost-effectiveness thresholds according to the authors in the majority of studies. In general, abatacept versus comparators after conventional DMARDS appear to have a higher cost effect ratio when compared to abatacept versus comparators after TNF failure. Other cost analyses are highly dependent on the country of origin and the comparator treatments used. 

## 4. Discussion

The disease and economic burden of RA remains substantial for the patients and health systems of developed economies worldwide. The continuously increasing therapeutic armamentarium provides clinicians with numerous options for the treatment of RA. Even when adopting the health economics point of view, it is safe to emphasize on the need for mutual patient-clinician agreement on the treatment options and strategy to be followed throughout the course of the disease, as recommended by an international task force on RA [3]. However, the context of “scarce resources versus infinite needs” in which health systems are obliged to operate nowadays (more than ever) necessitates that all options should be evaluated both in terms of their cost as well as in terms of their effectiveness, in order to rationalize the allocation of resources and achieve economic efficiency, that is, more and better “health” for the given resources. 

Placed in the previous line of thought, the present study aimed to review the existing economic evaluation evidence for abatacept, a recently approved agent for the treatment of RA [9]. For that purpose, a systematic literature review was performed in order to depict a thorough overview of the clinicoeconomic value of abatacept in the management of rheumatoid arthritis, compared to existing alternatives.

### 4.1. Cost Effectiveness of the Use of Abatacept following Conventional DMARDs

The criteria to initiate a biologic agent after failure of conventional DMARDs have been analyzed by several rheumatology bodies (ACR, EULAR, BSR) [6]. Usually, a biologic is started after failure to achieve a DAS-28 of <3.2 with conventional therapies [51]. The results of the systematic review on the use of abatacept after failure of DMARDs included studies mostly performed in American countries and in lesser cases in the European setting, evaluating the use of abatacept or other agents, after failure of methotrexate and taking into account a third party payer (social insurance) perspective.

In all 9 studies that were retrieved, abatacept was a cost-effective strategy against comparators after failure of methotrexate, although in varying degrees. This ranged from abatacept being a dominant strategy (i.e., more effective and less costly), versus etanercept, adalimumab, infliximab, and tocilizumab ([14], for the health system of Peru) and tocilizumab alone ([15], for the health system of Colombia), to borderline cost effective, the latter depending on the acceptability threshold that is used for local reimbursement decisions. 

In some cases of the referenced studies, abatacept led to reductions in the total costs of treatment accompanied (in all cases) by gains in Quality Adjusted Life Expectancy, thus leading to favorable economic evaluation results. 

Specifically, when compared to infliximab, abatacept proved to be associated with higher treatment costs, especially during the initiation phase [12, 14, 16, 17]. However, all of the previous studies concluded on the cost effectiveness of abatacept compared to infliximab, as these costs were offset by the increased number of QALYs gained. One study [17] estimated the offsetting of costs to take place at a maximum of around 16 months after initiation, claiming that abatacept appears to be an economically attractive, long-term treatment option in RA. Another study comparing the combination of abatacept and methotrexate with conventional DMARD treatment after failure of methotrexate alone also demonstrated that the abatacept strategy was able to offset the higher treatment costs, producing an ICER per QALY gained of *£*27,657. The indirect comparison to other biologic therapies, such as etanercept and adalimumab [13, 16], demonstrated the cost effectiveness of abatacept in active rheumatoid arthritis after inadequate response to conventional DMARDs. One study compared rituximab with abatacept [13]. Regarding RTX, ABA has an incremental cost-effectiveness ratio of S/75 493 per QALY gained.

### 4.2. Cost Effectiveness of the Use of Abatacept following TNF-a Inhibitors

The majority of retrieved results investigated the cost effectiveness of abatacept use after inadequate response or intolerance to one or more TNF-a antagonists (*N* = 21). Most studies focus on the comparison between abatacept and rituximab, either “head to head” or with the inclusion of other alternatives. This comparison is in favor of abatacept, since 10 studies report that abatacept is more cost effective or dominant versus rituximab and 4 state the opposite. However, it needs to be noted that 2 of the 4 studies in favor of rituximab are based on the (rather bold) hypothesis of equal efficacy across all agents under evaluation, thus selecting the cheapest one as the dominant option ([28] for the Brazilian healthcare setting and [29] for the Italian health care setting).

Other findings of interest include the improved cost effectiveness of abatacept compared to methotrexate in Hungary [31] and the UK [38], the association of abatacept with lower overall costs of treatment from a managed care perspective in the US [32], and the favorable cost-effectiveness results of abatacept in comparison to DMARDs in general [23, 25].

### 4.3. Abatacept and Its Value as Presented in Other Types of Pharmacoeconomic Analyses

A series of other economic evaluations of abatacept could not be classified to the previously mentioned major treatment pathways. Nevertheless, they provide some useful insights of the value of abatacept in the treatment of RA patients. Indicatively (a) the cost utility analysis by Wong et al. [41] (comparing methotrexate and abatacept regimens versus methotrexate and rituximab or methotrexate alone), which demonstrated that the combination of methotrexate and abatacept was well within the US cost-effectiveness threshold and (b) the analysis of abatacept versus sequential use of anti-TNF agents for the Canadian health care setting [7] which demonstrated that abatacept was highly cost effective.

Regarding the macroeconomics of the healthcare system, cost-of-illness analyses can be of great use to decision makers. In this field, Wong et al. have demonstrated lower acquisition and administration costs for abatacept [41] although a recent review [40] did not include the agent among those with the lowest cost per responder. Of particular interest is the study of Cole et al. [43] who showed that the use of abatacept can fully offset productivity losses for RA patients, a finding similar to that by Yuan et al. [42].

### 4.4. Limitations

Being performed in an environment with inherent uncertainty, economic evaluations of health interventions, especially in the rheumatoid arthritis, are characterized by a number of limitations points of discussion that should be addressed when interpreting the outcomes of any analysis. A limitation that applies for most of the economic evaluations presented here lies in the fact that the majority of them adopts a third party payer perspective, thus including only those costs that are relevant for a social security organization and not incorporating the so called “societal costs.” Third party payer perspective analyses are really useful when pricing or, especially, when reimbursement decisions must be supported; this is why they are currently acceptable according to most national and international guidelines for economic evaluations and performed more often than evaluations with a societal perspective. However, this way entails exclusion of a large fraction of indirect (non-health) costs, mainly productivity losses and costs of informal care, which in the case of RA is important, given the disabling nature of the disease.

Another issue that should be taken into account when interpreting the results of the present analysis lies within an inherent problem of economic evaluations, that is, the transferability of cost-effectiveness data among varying healthcare settings. Although guidelines on the best practices for economic evaluation and the uniformity of calculations do exist, variability in the organization, administration of care, and, thus, costs, between health systems, renders the caution on the interpretation of the results a necessity.

Other important limitations that specifically apply to this study are the exclusion of research presented in languages other than English and the lack of full paper articles for some of the research presented. Specifically, for the majority of the ISPOR studies, only abstracts were retrievable. So, there remains a (small) degree of uncertainty as to whether all the information available internationally regarding the value of abatacept has been in fact included in the present review and therefore, the findings have to be treated with caution. Future similar reviews will be able to identify full-text availability of the included research in abstract format. Finally, it has to be noted that one of the included studies [20] is a health technology assessment for the British National Institute for Health and Clinical Excellence (NICE), for which a small number of included studies may have been used in the analysis. The authors decided to include this HTA review, irrespective of this minor duplication, due to the significance of such a study. The review by Liu et al. [40] does not include any studies that have been identified and included in this analysis.

Rheumatoid arthritis is a disease with high prevalence and a significant social burden. Interventions with proven efficacy, such as abatacept, are an important addition to the clinician's armament. However, fiscal realities and scarce resources make cost-effectiveness data essential for decisions about treatment in the micro- (patient) as well as the macro- (healthcare system) level. The majority of data from the international research shows that abatacept is both clinically efficient and highly cost effective in the treatment of moderate to severe rheumatoid arthritis, especially in patients that have demonstrated inadequate response or intolerance to anti-TNF agents or DMARDs.

## Figures and Tables

**Table 1:** Cost effectiveness of abatacept versus other therapies in rheumatoid arthritis after failure of conventional DMARDs.

Origin and publication year	Type of publication	Source	Type of economic evaluation	Comparing treatments	Results	Comments
USA 2008 [10]	Research article	Pubmed	CEA	MTX versus MTX and ABA	ICER per QALY. Over a decade $47,910. Over a lifetime $43,041	Moderate to severe active RA after inadequate response to MTX.

Brazil 2008 [11]	Poster abstract	ISPOR	CEA	ABA versus MTX	Over the lifetime, ABA therapy was estimated to yield an average of 1.61 additional QALYs per patient (versus MTX alone) at a mean incremental cost of R$146.095/QALY (US$83,483, US$1 = R$1.75).	Moderate to severe active RA after inadequate response to MTX.

Colombia 2009 [12]	Poster abstract	ISPOR	CEA BIA	ABA versus TOC	A hypothetical cohort of 1,000 patients with RA-IR MTX followed for 20 years or until death, the mean direct medical costs per patient for ABA were U$132,654 (129,198–145,203), compared to U$283,753 (275,809–315,551) for TOC. For the group of subjects treated with ABA, 84% of these costs were associated with the drug; for TOC, 93% of the costs are associated with the drug. The mean numbers of life years were 29.27 (28.45–30.15) and 29.25 (28.43–30.13) for ABA and TOC, respectively. The mean numbers of QALYs (discounted) by ABA and TOC were 7.21 (7.02–7.42), and 7.15 (6.96–7.37), respectively.	After inadequate response to MTX, using ABA as the reference treatment, TOC provided less utility at a higher cost, being dominated by ABA.

Venezuela 2011 [13]	Poster abstract	ISPOR	CEA BIA	ABA versus INF	A hypothetical cohort of 1,000 patients with RA and IR MTX in Venezuela, followed for 10 years, resulted in mean treatment costs of U$5,126, U$57,824, and U$27,842 dollars, for MTX, ABA, and INF, respectively. Total direct medical costs (discounted) per patient were U$50,441 (48,819–52,448) for MTX, U$93,992 (89,366–98,982) for ABA, and $73,100 (68,539–81,877) for INF. The total QALYs gained (discounted) by MTX, ABA, and INF during the same period were 2.96 (2.89–3.03), 4.05 (3.85–4.30) and 3.26 (3.16–3.39), respectively. The Incremental Cost-Effectiveness Ratio was U$39,980 (36,649–45,011) for ABA compared to MTX compared to U$77,790 (62,369–98,124) per QALY gained with INF.	The use of ABA is more cost-effective than the use of INF, both compared to MTX, in patients with RA with IR MTX in Venezuela.

Peru 2011 [14]	Poster abstract	ISPOR	CEA	ABA versus other biologic DMARDs	The cost of treatment with ABA resulted in S/. 169 263 and its effectiveness was found to be 1.96 QALY. Regarding ETA, ADA, INF, and TOC, ABA was shown to be the most effective in terms of QALYs and the least expensive. Regarding RTX, ABA has an incremental cost effectiveness ratio of S/. 75 493 per QALY gained.	ABA was found to be dominant against ETA, ADA, INF, TOC, from the Health Social Security (EsSalud) perspective for the treatment of moderate to severe active RA after IR to MTX.

Colombia 2011 [15]	Poster abstract	ISPOR	CEA BIA	ABA versus INF	In a hypothetical cohort of 1,000 patients with RA-IR MTX, the costs of treatment for the first year for MTX were U$794 dollars, compared to U$16,659 for ABA and U$17,531 for INF, assuming dosages for average patients below 60 kg. Additional analysis with patients over 60 kg was included in the sensitivity analysis. After 10 years of followup the discounted total direct medical costs per patient were U$55,998 (54,354–57,776) for MTX, U$99,888 (94,694–104,437) for ABA, and $79,174 (75,795–83,899) for INF. The total numbers of QALYs gained (discounted) by MTX, ABA, and INF were 2.88 (2.79–2.95), 3.94 (3.79–4.09), and 3.17 (3.09–3.27), respectively. The calculated ICERs for ABA and INF compared to MTX were U$37,513 (35,221–39,909) and U$75,873 (62,825–103,132) per QALY gained, respectively.	In patients with RA-IR MTX in Colombia, the use of ABA is more cost effective than the use of INF, both compared to MTX.

UK 2010 [16]	Poster abstract	ISPOR	CEA	ABA with MTX followed by a sequence of DMARDs was compared against a sequence of cDMARDs	ABA with MTX was estimated to yield 1.09 QALYs per patient (6.42 versus 5.33) over lifetime, compared to DMARDs. The total lifetime costs associated with ABA with MTX were *£*110,094 and total costs for cDMARDs were *£*79,933 resulting in an ICER of *£*27,657 per QALY gained. Sensitivity analysis confirmed the robustness of the model findings.	This study has demonstrated that ABA with MTX is a cost effective treatment option compared to cDMARDs for patients with RA after an inadequate response to MTX.

Canada 2010 [17]	Poster abstract	ISPOR	CEA	MTX versus ABA, ADA, INF, and ETA	ABA has a cost-effectiveness ratio of approximately $93,000 per QALY gained versus MTX, comparable with those of ETA ($96,000) and ADA ($112,000) and much lower than that of INF ($171,000). At willingness-to-pay between $80,000 and $97,000, ABA is the most cost-effective option. Results were most sensitive to the assumption of the threshold for clinically meaningful HAQ improvement at 6-month and applied time horizon.	ABA offers a valuable therapeutic option for the treatment of moderate-to-severe active RA in patients with inadequate response to one or more DMARD therapies.

Italy 2011 [18]	Poster abstract	ISPOR	CCA	ABA versus INF	In Italy, the annual trial-based costs per remitting/LDAS patient were 70,259/37,219 for ABA versus 85,547/46,592 for INF. In the initiation phase, costs per patient-month in remission/LDAS were 11,028/6,020 for ABA versus 8,347/4,173 for INF. ABA showed lower costs per patient-month in remission/LDAS in the maintenance phase 5,046/2,673 versus 5,500/2,996 for INF. Real-life maintenance costs per month in remission/LDAS were 5,347/2,832 for ABA versus 7,210/3,927 for INF. Higher initiation cost for ABA to achieve remission/LDAS would be offset at 14.6/16.1 months during real life.	Findings suggest a lower cost consequence for ABA during the maintenance phase and its real-life extrapolation. ABA is a sustainable, safe, and economically attractive biologic for the long-term treatment of RA when compared to INF.

**Table 2:** Cost effectiveness of abatacept versus other therapies in rheumatoid arthritis after failure of a TNF antagonist.

Origin and publication year	Type of publication	Source	Type of economic evaluation	Comparing treatments	Results	Comments
Finland 2012 [19]	Research article	Pubmed	CEA	ABA versus RTX in sequential biologic regimes	Mean costs per day in RS and LDAS were, respectively, €829 and €428 for the biologic sequence composed of ADA-ABA-ETA, €1292 and €516 for the sequence ADA-RTX-ETA, €829 and €429 for the sequence ETA-ABA-ADA, €1292 and €517 for the sequence ETA-RTX- ADA, €840 and €434 for the sequence INF-ABA-ETA, and €1309 and €523 for the sequence INF-RTX-ETA.	In moderate or severe RA when a clinical response to a first TNF-blocker, (ETA, ADA, or INF), is insufficient, the treatment sequences including ABA as the second biologic option appear more cost effective than those including RTX.

UK 2011 [20]	Review	Pubmed	CEA	RTX, ABA, INF, ETA, ADA	ICER per QALY: ABA *£*38,600. ADA *£*34,300. RTX *£*21,200. ETA *£*38,800. INF *£*36,200.	Tx after failure of a TNF inhibitor. ABA versus RTX ICER > *£*100,000.

Italy 2011 [21]	Research article	Pubmed	CEA	ABA versus RTX and ABA versus sequential anti-TNF agents (>2)	ICER per day in LDAS: ABA (after one anti-TNF agent) more cost effective versus RTX (€376 versus €456). ABA used (after two anti-TNF agents) more cost effective versus sequential anti-TNF use (€642 versus €1164).	Moderate-to-severe active RA after an insufficient response to anti-TNF agents. ABA appears to be more cost effective than other compared regimes.

Finland 2010 [22]	Research article	Pubmed	CUA	ADA, ABA, ETA, INF, RTX	ICER per QALY gained: €30,248 for RTX compared with BSC. €50,941, €50,372, €36,121, and €67,003 per QALY gained for adding ADA, ETA, INF, and ABA to the RTX strategy, respectively.	In severe RA after insufficient response to anti-TNF agents. Treatment with RTX is a cost-effective treatment strategy in RA patients in Finland.

Spain 2011 [23]	Research article	Pubmed	CEA	ABA versus RTX as second biological option	ICER per day in LDAS: ABA more cost effective than RTX (€427 versus €508).	In patients with an insufficient response to anti-TNF agents.

UK 2011 [24]	Poster abstract	ISPOR	CEA	ABA versus conventional DMARDs	ABA was estimated to yield 1.06 additional quality-adjusted life years (QALYs) per patient (3.28 versus 2.22) over a lifetime, compared to conventional DMARDs. The total lifetime costs associated with ABA were *£*46,522 and total costs for conventional DMARDs were *£*17,025, resulting in an incremental cost-effectiveness ratio (ICER) of *£*27,936 per QALY gained. Probabilistic and univariate sensitivity analyses confirmed the robustness of our findings.	Abatacept is a cost-effective treatment option for patients with RA after the failure of a first anti-TNF in the UK.

France 2010 [25]	Research article	Pubmed	CEA	ABA versus RTX as second biological option	ICER per TEND: ABA more cost effective than RTX (€278 versus €303).	In patients with inadequate response to the first anti-TNF agents (ETA).

US 2008 [26]	Research article	Pubmed	CEA	DMARDs versus DMARDs and ABA	ICER per QALY. Over a decade $50,576. Over a lifetime $45,979.	Treatment after failure of a TNF inhibitor. Moderate to severe active RA.

Canada 2008 [27]	Poster abstract	ISPOR	CEA	ABA or RTX versus placebo	Relative to placebo, ABA therapy was estimated to yield an average of 1.07 additional QALY per patient, at a mean incremental cost of $45,875; RTX therapy was estimated to yield an average of 0.94 (95% CI = 0.82, 1.05) additional QALY per patient, at a mean incremental cost of $51,101. Lifetime treatment with ABA was estimated to be a dominant strategy compared with RTX, primarily due to lower treatment costs and better HAQ improvement.	ABA therapy would be economically attractive from a Canadian payer perspective.

Brazil 2008 [28]	Poster abstract	ISPOR	CMA BIA	RTX versus ABA, INF, ADA, ETA	RTX therapy resulted in a total annual cost of R$46,388 per patient. Total annual costs per patient for the comparators were R$79,394 for INF, R$90,831 for ADA, R$120,351 for ETA, and R$77,118 for ABA. In the BIA, RTX therapy resulted in total savings of R$94,201,413 in 5 years considering the population in the private health care system only. Results were sensitive to dosage schedule (RTX, INF, and ABA) and drug costs.	In patients with IR to anti-TNF. The study assumed equal efficacy and assessed the total cost of RTX therapy in comparison with INF, ADA, ETA, and ABA. RTX is a dominant alternative.

Italy 2008 [29]	Poster abstract	ISPOR	BIA	ABA, RTX	The simulated effect of a progressive replacement (16%, 50%, and 100% at years 1, 5, and 10, resp.) of the current strategy with the two new strategies yielded the following results. RTX therapy induces a decrease of costs of 1.16 million Euro (−7.1% with respect to current strategy), 4.91 (−7.7%), and 4.94 (−5.3%) at years 1, 5, and 10, whereas ABA therapy induces an increase of 0.6 million Euro (+3.7%), 5.43 (+8.5%), and 20.62 (22.2%).	When only direct medical costs were considered, the direct comparison between the two new strategies, RTX results less expensive.

France 2008 [30]	Poster abstract	ISPOR	CEA	ABA (as second biologic agent—Strategy A) versus RTX (as second biologic agent—Strategy B).	Using the LDAS endpoint, Strategy A was significantly more efficacious over 2 years versus Strategy B, with 134 versus 107 TEND under LDAS. Mean cost-effectiveness ratios showed significantly lower overall medical costs per TEND under LDAS with Strategy A versus Strategy B (€212 versus €234). Using the remission endpoint, Strategy A was significantly more efficacious over 2 years versus Strategy B, with 61 versus 37 TEND under remission. Mean cost-effectiveness ratios showed significantly lower overall medical costs per TEND under remission with Strategy A versus Strategy B (€446 versus €642; *P* < 0.01).	Moderate to severely active RA and an inadequate response to anti-TNF therapy. When used as the second biologic agent after an inadequate response to one anti-TNF agent, ABA appears significantly more efficacious and cost effective than RTX.

Italy 2008 [31]	Poster abstract	ISPOR	CUA	ABA versus INF, ADA, ETA	ABA versus anti-TNF therapies was estimated to yield 0.66 additional QALYs per patient at an incremental cost of €10,096.40, based on a 20 years' time horizon. Cost per QALY gained was €15,278.20. The acceptability curve showed that ABA has a likelihood of 100% to be cost-effective in comparison to anti-TNFs with willingness-to-pay threshold of €30,000.00.	Compared to anti-TNF therapies, ABA is cost effective in moderately to severely active RA and an insufficient response or intolerance to anti-TNF therapies.

Hungary 2008 [32]	Poster abstract	ISPOR	CUA	ABA versus MTX, DMARDs and cycled anti-TNF	ABA was cost effective compared to MTX, yielding 0.57 additional QALY at cost of 2.03 million HUF with an ICER of 3.6 million HUF/QALY. From the Hungarian health insurance perspective, ICER was 4.4 million HUF/QALY gained. Compared to cycled anti-TNFs, ABA was dominant, with a QALY gain of 0.48 and estimated savings of HUF 731113. From the Hungarian health insurance perspective, the savings were 479 815 HUF. The results are robust to extensive sensitivity analyses.	RA patients with an inadequate response to anti-TNFs: A simulation cost-utility model based on disease progression expressed in HAQ disability index score change was developed to enroll patients corresponding to the patients of the ATTAIN clinical trial. This cost-utility analysis was conducted using a societal perspective, including all costs (direct and indirect). The results of this cost-utility assessment suggest that ABA is cost-effective compared to MTX and to cycled anti-TNFs in Hungary.

USA 2010 [33]	Poster abstract	ISPOR	Cost of therapy	ABA, RTX, INF	Total RA-related health care costs (RTX = $26,783; ABA = $24,344; INF = $27,053; *P* = 0.1249), RA medication costs (RTX = $19,973; ABA = $19,000; INF = $20,763; *P* = 0.1840), and Index biologic costs (RTX = $17,638; ABA = $16,233; INF = $17,845; *P* = 0.1256).	From a U.S. commercial payer perspective No significant difference in one-year RA-medication costs or total RA-related costs between patients starting RTX, ABA, or INF was identified. ABA was associated with lowest costs.

UK 2009 [34]	Poster abstract	ISPOR	CEA	Assuming an IR to the 1st anti-TNF agent, Sequence 1 included ETA-ABA-ADA and Sequence 2 ETA-RTX-ADA. Assuming an IR to 2 anti-TNF agents, Sequence 3 included ETA-ADA-ABA and Sequence 4 ETA-ADA-INF.	There were 6-month medical costs (excluding biologic drug costs) estimated at *£*1047 (Standard Deviation (SD) 332) for managing patients in LDAS and at *£*2650 (SD 963) for moderate-to-high disease activity. Over 2 years, Sequence 1 appeared more efficacious (92 days in LDAS) versus Sequence 2 (82 days in LDAS), with cost-effectiveness ratios of *£*281/day in LDAS versus *£*289/day in LDAS, respectively. Sequence 3 appeared more efficacious (43 days in LDAS or) versus Sequence 4 (32 days in LDAS), with cost-effectiveness ratios of *£*603/day in LDAS versus *£*809/day in LDAS, respectively.	The results of this simulation model suggest that, for achieving LDAS, sequences including ABA after an IR to at least 1 anti-TNF agent appear more cost-effective than similar sequences including RTX or cycled anti-TNF agents.

Germany 2009 [35]	Poster abstract	ISPOR	CEA	Four sequential biologic strategies composed of anti-TNF agents versus ABA or RTX	Over 2 years, the sequence with ABA after 1 anti-TNF agent appeared the most effective and cost effective versus use after 2 anti-TNF agents (633 versus 1067/day in LDAS and 1222 versus 3592/day in remission) and versus a similar sequence using RTX (633 versus 728/day in LDAS and 1222 versus 1812/day in remission). The sequence using a 3rd anti-TNF agent was less effective and cost effective than the same sequence using ABA (2000 versus 1067/day in LDAS and 6623 versus 3592/day in remission).	The results suggest that in patients with an IR to at least one anti-TNF agent, biologic sequences including ABA appear more efficacious and cost-effective than similar sequences including RTX or cycled anti TNF agents.

Turkey 2009 [36]	Poster abstract	ISPOR	CEA	Six treatment strategies using three successive biologic agents ETA, ADA, INF, ABA, RTX	Considering an IR to 1 anti-TNF agent, the sequence ETA-ABA-ADA was more efficacious and cost effective (102 days in LDAS; 496 TL per day in LDAS) over 2 years than the sequence ETA-RTX-ADA (82 days in LDAS; 554 TL per day in LDAS or 81 days in LDAS; 563 TL/day in LDAS based on RTX current reimbursement status). Considering an IR after 2 anti-TNF agents, the sequences ETA-ADA-ABA and ETA-INF-ABA were more efficacious and cost effective (64 days in LDAS for both; 841 and 826 TL/day in LDAS, respectively) over 2 years than a sequence of anti-TNF agents only (32 days in LDAS; 1480 TL per day in LDAS).	Sequences including ABA after an IR to one anti-TNF agent are more efficacious and cost-effective versus similar sequences including RTX. Sequences including ABA after an IR to 2 anti-TNF agents also appear more effective and cost effective than similar sequences composed of anti-TNF agents only.

Canada 2009 [37]	Poster abstract	ISPOR	CEA	ABA or RTX versus standard therapy	The introduction of RTX following failure of one biologic resulted in a gain of 0.443 QALYs at an additional total cost of $3710 resulting in an ICER of $8380/QALY. The introduction of ABA following failure of one biologic resulted in a gain of 0.387 QALYs at an additional total cost of $18,588 resulting in an ICER of $48,000/QALY.	RTX is economically attractive from a Canadian payer perspective and is a cost-effective treatment option over ABA when compared in the studied population.

UK 2007 [38]	Poster abstract	ISPOR	CUA	ABA versus RTX	Annual drug acquisition and administration costs were lower for RTX compared to ABA. Discounted total lifetime direct NHS costs were *£*46,570 and *£*63,055 for the RTX and ABA groups, respectively. RTX generated a discounted cost saving of *£*16,485 per patient due to reduced drug acquisition and administration costs. Total QALYs were estimated as 3.879 and 3.812 for RTX and ABA, respectively.	The model predicted that RTX dominated ABA for RA patients who have failed one previous TNF inhibitor therapy, with higher estimated QALYs and lower NHS costs.

UK 2007 [39]	Poster abstract	ISPOR	CUA	ABA versus MTX	Compared to MTX, ABA treatment results in 1.6 additional QALYs at an additional cost of *£*40,371, giving an ICER of *£*25,395/QALY, a value in line with other biologic treatments recommended for use in the UK.	Compared to MTX, ABA is a cost-effective treatment.

**Table 3:** Other pharmacoeconomic studies of abatacept in rheumatoid arthritis.

Origin and publication year	Type of publication	Source	Type of economic evaluation	Comparing treatments	Results	Comments
US 2012 [40]	Review	Pubmed	Cost of treatment	ABA, ADA, CER, ETA, GOL, INF, RTX, TOC	In RA, biologics with the lowest 6-month costs per responder were ADA ($27,853; 95% CI $19,284–40,270), ETA ($29,140; 95% CI $14,170–61,030), and TOC ($31,363; 95% CI $14,713–64,232).	Cost per responder was estimated for each biologic as projected per patient drug costs (2011 US$) divided by response rate difference.

US 2011 [41]	Research article	Pubmed	Cost of treatment	ABA, INF, RTX	Total drug associated costs per surgery visit were $2,828, $1,827, and $6,076; and the costs of IV administration were $224, $171, and $390, respectively, for INF, ABA, and RTX.	ABA acquisition and administration costs lower than comparator treatments.

US 2010 [42]	Research article	Pubmed	CUA	MTX versus ABA/MTX and RTX/MTX	ICER per QALY: $47,191 for ABA/MTX and $54,891 for RTX/MTX versus MTX alone.	Third party payer perspective. Indirect costs. RTX dominated by ABA.

Canada 2012 [7]	Research article	Pubmed	CEA	ABA versus sequential use of anti-TNF agents	As first biologic agent, ABA appears significantly more cost effective compared to the sequential use of anti-TNF agents (*P* < 0.001). ABA, as second biologic agent, appears significantly more cost effective compared to the sequential use of anti-TNF agents (*P* < 0.001).	ABA after failure of DMARDs and after failure of one anti-TNF agent.

US 2008 [43]	Research article	Pubmed	Cost analysis relative to QoL	ABA versus placebo	ABA led to greater reduction in medical expenditure over time in MTX failure ($152 lower) and anti-TNF failure patients ($122 lower) compared with placebo. Likewise, significantly more reduction in likelihood for current and future job loss was achieved with ABA.	Two RCTs. One with patients who have not responded to MTX and the other with patients unresponsive to anti-TNF agents.

US 2008 [44]	Research article	Pubmed	Cost analysis relative to lost productivity	ABA versus no treatment	The lost productivity cost of RA for a firm of 10,000 was $1.69 million. In the base case analysis 37% of the acquisition cost of abatacept was offset by reductions in the cost of RA-related productivity losses. In some industry groups (Utilities and Finance) and in models that included presenteeism, reductions in lost productivity costs exceeded the abatacept cost.	Productivity losses for a private firm of 10,000 due to employee absences and reduced effectiveness at work because of rheumatoid arthritis.

Canada 2008 [45]	Poster abstract	ISPOR	CEA	ABA versus MTX, DMARDs, placebo, INF	(1) AIM: on a lifetime basis, ABA was estimated to yield an average of 1.4 additional QALYs per patient versus MTX at a mean incremental cost of $54,331; the estimated CE of ABA was $39,604 per QALY gained. (2) ATTAIN: ABA yielded an average of 1.2 additional QALY versus oral DMARDs alone at a mean incremental cost of $50,141; estimated CE of ABA was $42,021per QALY. (3) Trial 043: relative to placebo, ABA yielded an average of 1.58 additional QALY at ICER of $58,351. ICER of ABA was $37,094 per QALY; INF yielded an average of 1.24 additional QALY at a mean incremental cost of $53,305; incremental CE of INF was $43,247 per QALY. Relative to infliximab, the incremental CE of ABA was about $14,841 per QALY.	Active RA and failure to MTX or anti-TNF. Simulation model to depict progression of functional disability over time. Functional disability was expressed in terms of HAQ-DI. The model separately estimated CE using data from three phase III clinical trials ABA is cost effective in patients with active RA and inadequate response to DMARD or anti-TNF therapy and also highly cost effective relative to INF.

Italy 2011 [46]	Poster abstract	ISPOR	BIA	The sequence including ABA (ABA-IFX-RTX) as first line was compared with: ETN-IFX-RTX; ADA-IFX-RTX	Italian target population was estimated in about 7000 RA patients. At the end of the third year patients still on first biologic drug were 5670, 4610, and 4680 in the sequence with ABA, ETN, and ADA. Patients in ACR I or II were 6240, 6160, and 6000, respectively. The annual cost at the third year was 47.0 million, 48.5 million, and 47.8 million for the sequence with ABA, ETA, and ADA, respectively.	The use of ABA as first biologic line treatment for RA showed to provide better control of the disease along with a positive impact in total costs, when compared with traditional sequences based on anti-TNFa in Italy.

Brazil 2009 [47]	Poster abstract	ISPOR	BIA	All 1st line biologic therapies	Total annual costs were $Brz81,021 for TOC, $Brz92,789 for ETA, $Brz98,541 for ADA, $Brz105,283 for INF, and $Brz85,020 for ABA. Based on the change in the forecast, total savings for a period of 5 years were $Brz1,573,902 for each 100 treated patients, when comparing the group of patients received TOC to the group of patients that did not receive TOC.	Findings suggest TOC offers potential costs reductions in the private healthcare system in Brazil.

Brazil 2009 [48]	Poster abstract	ISPOR	BIA	All 1st line biologic therapies	Total annual costs were $Brz47,566 for TOC, $Brz50,785 for ETA, $Brz53,909 for ADA, $Brz58.603 for INF and $Brz50,048 for ABA. Based on the change in the mix of treatment forecast, total savings for a period of 5 years were $Brz674.527 for each 100 treated patients, when comparing the group of patients that received TOC to the group of patients that did not receive TOC.	Findings suggest TOC has the potential to offer costs reductions in the public healthcare system in Brazil.

Brazil 2008 [49]	Poster abstract	ISPOR	BIA	ABA versus current practice	In the first year of inclusion there is an increase in costs of R$3,085,711 (US$1,763,263). In the second and third years there are significant savings on budgets, R$97,593,971 (US$55,767,983) and R$129,458,357 (US$73,976,204), respectively.	The incorporation of ABA into the NPEM is cost saving to the Brazilian Public Health System, saving R$129,458,357.00 (US$73,976,204) after the third year.

USA 2008 [50]	Poster abstract	ISPOR	Cost of treatment	ABA versus INF	The estimated annual drug plus infusion administration cost of first and subsequent biologic therapy was $13,354 and $14,465 for ABA and $16,608 and $23,913 for INF, respectively.	Patients treated with INF experienced an increase in dosage and/or dosing frequency, resulting in an increase in real world treatment costs. Patients treated with ABA showed no considerable increase in dose or dosing frequency from first to last infusion.

## Abbreviations

ABA: Abatacept
ADA: Adalimumab
ANA: Anakinra
CER: Certolizumab
CRD: Centre for reviews and dissemination
DARE: Database of abstracts of reviews of effects
DMARDs: Disease modifying antirheumatic drugs
ETA: Etanercept
GOL: Golimumab
HTA: Health technology assessment
ICER: Incremental cost-effectiveness ratio
INF: Infliximab
IR: Inadequate response
ISPOR: International Society for Pharmacoeconomics and Outcomes Research
MTX: Methotrexate
NHS EED: National Health Service Economic Evaluation Database
NICE: National Institute for Health and Clinical Excellence
NSAIDs: Nonsteroidal anti-inflammatory drugs
TNF: Tumour necrosis factor
RA: Rheumatoid arthritis
RTX: Rituximab
TOC: Tocilizumab
QALY: Quality adjusted life year.
